# Erosion of Conserved Binding Sites in Personal Genomes Points to Medical Histories

**DOI:** 10.1371/journal.pcbi.1004711

**Published:** 2016-02-04

**Authors:** Harendra Guturu, Sandeep Chinchali, Shoa L. Clarke, Gill Bejerano

**Affiliations:** 1 Department of Electrical Engineering, Stanford University, Stanford, California, United States of America; 2 Department of Pediatrics (Genetics), Stanford University, Stanford, California, United States of America; 3 Department of Computer Science, Stanford University, Stanford, California, United States of America; 4 Department of Genetics, Stanford University, Stanford, California, United States of America; 5 Department of Developmental Biology, Stanford University, Stanford, California, United States of America; Rutgers University, UNITED STATES

## Abstract

Although many human diseases have a genetic component involving many loci, the majority of studies are statistically underpowered to isolate the many contributing variants, raising the question of the existence of alternate processes to identify disease mutations. To address this question, we collect ancestral transcription factor binding sites disrupted by an individual’s variants and then look for their most significant congregation next to a group of functionally related genes. Strikingly, when the method is applied to five different full human genomes, the top enriched function for each is invariably reflective of their very different medical histories. For example, our method implicates “abnormal cardiac output” for a patient with a longstanding family history of heart disease, “decreased circulating sodium level” for an individual with hypertension, and other biologically appealing links for medical histories spanning narcolepsy to axonal neuropathy. Our results suggest that erosion of gene regulation by mutation load significantly contributes to observed heritable phenotypes that manifest in the medical history. The test we developed exposes a hitherto hidden layer of personal variants that promise to shed new light on human disease penetrance, expressivity and the sensitivity with which we can detect them.

## Author Summary

A central goal of personal genomics is to interpret an individual’s genome to identify variants that confer disease risk, an aim that has far-reaching implications for personalized, precision medicine. Here, we leverage next generation sequencing, health records, and functional genome annotations to develop statistical methods that predict disease risk from a single genome. Motivated by the fact that about 90% of genome-wide association study disease-associated variants lie in the non-coding genome, we identify personal variants that mutate conserved transcription factor binding sites. To identify if such non-coding personal variants collectively dysregulate a key biological process, we employ the enrichment analysis tool GREAT to identify if a person’s noncoding mutations are over-represented in the regulatory domains of genes involved in a common biological pathway. Notably, in five unrelated genomes we analyzed, the most statistically significant, seemingly dysregulated pathway is indicative of that person’s medical history, ranging from neuropathy to heart disease. Statistical analysis confirms that associations from our predicted pathway to an individual’s medical record are rigorous and significant in the context of the un-phenotyped, race-matched 1,000 Genomes cohort. As such, we present a novel method that leverages the contribution of multifactorial non-coding variation to predict disease risk in individual genomes.

## Introduction

The advent of high-throughput genotyping spurred the rise of genome-wide association studies (GWAS) aimed at identifying the basis of genetic diseases. GWAS variants, over 90% of which have been found to localize outside of protein-coding sequences [1], and the growing body of non-coding genome annotations have helped improve our understanding of the genetic basis of diseases by shifting the focus from protein coding and copy number variations [2–4], to the non-coding genome. Though GWAS have been instrumental in suggesting a gene regulatory component to human disease susceptibility [5,6], they have been plagued by the “missing heritability problem”, which observes that loci detected by GWAS in general only explain a small fraction of the genetic variance responsible for phenotype [3,7].

Suggested models of genetic variance responsible for the “missing heritability problem” include “the infinitesimal model”–a large number of small effect common variants and “the rare allele model”–a large number of large-effect rare variants [7]. In the case of the infinitesimal model, the missing heritability can be explained due to additive or epistatic interactions between variants rather than independent polymorphisms [8]. But, selecting and evaluating all sets of variants results in a combinatorial explosion of sets that we are currently statistically underpowered to evaluate.

In this work, we will show how to not only successfully avoid the combinatorial explosion, but also simultaneously address the crucial role of additive and epistatic noncoding variation in human disease. Specifically, we develop a novel statistical framework to identify putatively deleterious noncoding variation in personal genomes that *en masse*, confers disease risk by dysregulating key genes involved in a common biological process.

A central role of the non-coding genome lies in *cis*-regulation of gene expression. GREAT (Genomic Regions Enrichment of Annotations Tool) is a tool commonly used to address the functional enrichment of a set of *cis*-regulatory genomic regions [9]. GREAT tests whether an arbitrary set of genomic regions, most of which are thought to regulate the expression of nearby genes, congregate next to genes of particular functions or pathways. GREAT assigns different genes variable length gene regulatory domains, accounts for distal regulatory elements and rewards observing multiple elements next to the same gene–reflecting observed properties of vertebrate gene regulation. GREAT has been shown to be superior to gene based tests (following the one probe—one gene paradigm of transcript analysis) in analyzing different types of ChIP-seq and related data [9].

Since we are interested in identifying disease-associated noncoding variation to interpret personal genomes, we asked whether disease-associated noncoding mutations would be functionally enriched for key biological pathways using GREAT. First, we subjected noncoding GWAS significant SNPs associated with several phenotypes ranging from Crohn’s disease to fasting glucose traits to GREAT (S1 Table). Not all GWAS tag-SNPs are themselves causal, but because they lie in proximity to the causal mutation, we can assume that GREAT will in most cases associate the tag-SNP with the same affected gene/s it would associate the underlying causal mutation. For example, if we subject the 40 non-exonic non-linked GWAS SNPs associated with cholesterol levels to GREAT analysis, the topmost enriched term (*P* = 3 x 10^−5^) in the entire GO ontology is genes involved in “abnormal circulating cholesterol level”. We show similar results across several different GWAS sets in S1 Table. In each case, we see that the non-linked tag-SNPs are most enriched next to genes of functional categories strikingly relevant to the assayed phenotype, providing *in-silico* assurances for the quality of the study and the validity of a GREAT analysis, but also suggesting that multiple of these mutations may accumulate in afflicted individuals. As such, we hypothesized that much more signal may hide in *cis*-regulatory variants beyond what GWAS may reveal.

The coherence of target gene enrichment for GWAS variants suggests additive and/or epistatic effects of variations to confer phenotype. Modeling such interactions is generally limited to heuristic search of pairs due to the high computational requirement and lack of statistical power [10]. The statistical power for identifying causal variants is further weakened in non-coding regions due to most variants resulting from neutral evolution of the genome [11]. Thus, to obtain a high quality set of variants on which GREAT can be applied we require a method of obtaining a set of functionally relevant noncoding variants without enumerating all possible sets.

To obtain a set of functionally relevant, putatively deleterious noncoding variants, we make use of transcription factor (TF) binding site prediction. Novel high throughput technologies, such as HT-SELEX and Protein Binding Microarrays, are revealing the precise DNA binding preferences of the majority of human transcription factors [12,13]. Using these preferences to predict TF binding in a single genome is notoriously hard. However, if one is willing to predict only a subset of binding sites, namely those conserved through evolution, one can then predict the existence of a binding site only if one sees the site in orthologous locations in a number of different mammals [14]. Such a scheme will naturally miss many evolutionarily newer binding sites, but, as we and others have shown, those conserved binding sites that we do predict are predicted with great precision and are useful for downstream analysis such as functional enrichment and protein complex prediction [14–16].

As shown in the GREAT paper, while a ChIP-seq experiment reveals that a TF binds non-specifically to many genomic locations, the strongest GREAT gene enrichment reflects the process or function the TF is regulating, highlighting the subset of binding sites involved in the regulatory process [9]. Previously, in our binding site prediction (PRISM) paper, we predicted the conserved subset of binding sites of a given TF motif and subjected this set, in place of ChIP-seq peaks, to GREAT analysis. In many cases, such as for transcription factors REST, GABPA, SRF, and STAT3, such analysis revealed multiple functional contexts in which the TF was involved without requiring a cell-type matched TF ChIP-seq experiment [14].

Additionally, in our previous binding site prediction work, we intersected our conserved binding site predictions with GWAS tag SNPs. To maximize the chance a GWAS tag SNP was indeed the functional, causal mutation, we set out to search for the following: A GWAS tag SNP overlapped by a conserved binding site prediction, such that: 1) the two observed alleles significantly differed in the predicted TF’s ability to bind to the motif, and 2) the TF we predict to bind has been previously implicated in the GWAS phenotype. In our paper, we highlighted only five such predictions (Table 1 in [14]). One striking example, in the context of prostate cancer, is our prediction that a GWAS risk allele at 6q22 modifies the conserved binding site of HOX13, thus modifying the expression of the downstream RFX6 gene. Our prediction was later beautifully experimentally validated by Taipale and colleagues, illustrating the utility of PRISM predictions in assessing the impact of noncoding variation on disease [17].

As exemplified above, the confluence of binding site prediction with PRISM and functional assessment of *cis*-regulatory regions with GREAT suggests a potent combination to understand the role of noncoding variation in disease. Accordingly, in this study we looked at personal genomes, tallied all the locations where the individual carries a SNP that disrupts an evolutionarily conserved binding site, and asked (using GREAT) which biological function or process these mutations aggregate next to most. Guided by our hypothesis that a pathway with the most unexpected mutational load may contribute to a person’s medical history, we then assessed our pathway predictions for relevance to the person’s health record.

## Results

**Fig 1:**
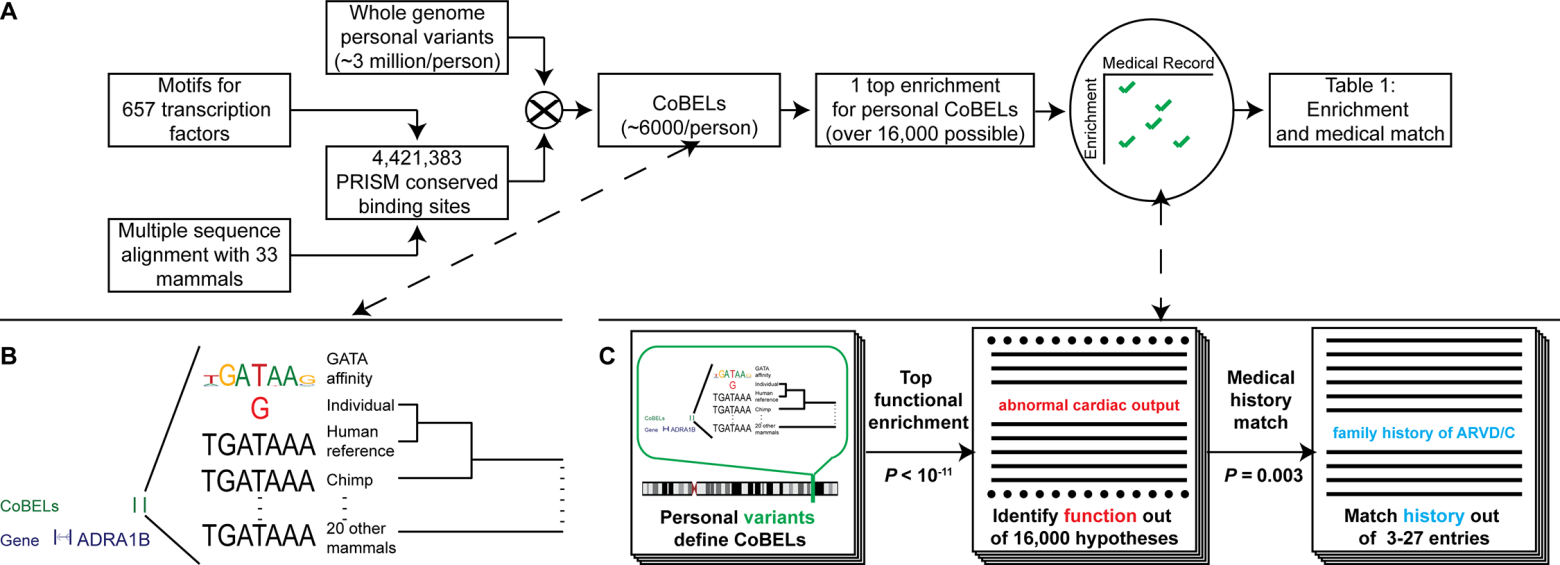
Schematic of conserved binding site eroding loci method. (A) Method for inferring conserved binding site eroding loci (CoBELs) and hypothesizing functional consequences of erosions. (B) Conserved binding site eroding loci (CoBELs) are human reference transcription factor binding sites, conserved across multiple mammals, that are disrupted by a sequenced individual’s derived variant. Shown is a CoBEL upstream of ADRA1B contributing to the Quake genome “abnormal cardiac output” prediction in Table 1. (C) Conserved binding site eroding loci (CoBELs) are checked for enrichment of function and the functional phenotypes are matched to medical histories via literature survey. Each step is evaluated for statistical significance (see text).

Using a large library of unique high quality binding motifs for 657 different transcription factors, covering all major human DNA binding domain families and a multiple alignment of 33 primates and mammals, we first predict cross-species conserved binding sites present in the reference human genome (see Materials and Methods). We then examine the genetic variants of a human individual against the reference genome. We focus on the subset of variants (heterozygous or homozygous) that overlap conserved binding site predictions. From these, we pick only variants where the human reference base is identical to its chimpanzee orthologous base (and thus most likely ancestral), and the individual variant base differs from both. Finally, of these we keep only the binding sites where the individual (derived) variant is predicted to significantly decrease binding affinity compared to the ancestral base–we call these conserved binding site eroding loci, or CoBELs (see Fig 1 and Materials and Methods).

We downloaded from UCSC whole genome variant files for all four individuals for whom public medical history summaries are also available: Stephen Quake [18], and three individuals from the personal genome project (PGP10) [19]. An additional file was obtained for James Lupski [20]. We then compared each separately to the reference genome to obtain 6,321 CoBELs for Stephen Quake, 5,291 for George Church, 5,775 for Misha Angrist, 5,861 for Rosalynn Gill, and 6,447 for James Lupski (S4–S8 Tables).

Because CoBELs weaken conserved ancestral binding sites, we asked whether an individual’s set is found preferentially next to genes encoding any particular function, and if so, whether this function relates to the individual’s medical history (Fig 1C). GREAT, as described earlier, is an approach devised specifically to assess enriched functions within a set of genomic regions thought to regulate the adjacent genes [9] by associating with each gene in the genome a variable length regulatory domain, bracketed by its two neighboring genes. GREAT also holds a large body of knowledge about gene functions and phenotypes–here we use over 1.1 million such gene annotations (see Materials and Methods). For a given set of CoBELs, GREAT iterates over 16,000 different biological functions and phenotypes, asking whether CoBELs are particularly enriched in the regulatory domains of genes of any particular function. For example, 33 genes in the human genome are annotated for “abnormal cardiac output”. Their GREAT assigned regulatory domains cover 0.45% of the genome. Of the 6,321 Quake CoBELs, 28 (0.45%) are expected in the regulatory domains of these 33 genes by chance, but 57 CoBELs, over twice as many, are in fact observed. To determine statistical significance, GREAT computes two statistics for this enrichment, and corrects them for multiple hypothesis testing (see Materials and Methods).

Prominent in Stephen Quake’s medical records is a family history of arrhythmogenic right ventricular dysplasia/cardiomyopathy, including a possible case of sudden cardiac death [18]. Strikingly, when Quake’s set of CoBELs is analyzed using GREAT, the top phenotype enrichment (using default parameter settings, optimized for inference power in the original GREAT paper [9]) is “abnormal cardiac output” (57 CoBELs, false discovery rate Q = 1.69 x 10^−4^). This enrichment is suggestive of susceptibility to heart diseases responsible for reduced cardiac output [21]. Meaningful associations between CoBELs and personal medical records are in fact observed for all five genomes (Table 1 and S9–S13 Tables).

**Table 1:** Top predicted phenotype and matching medical phenotype. The set of conserved binding site eroding loci (CoBELs) for each individual is searched for the most significant congregation of binding site erosion events next to a group of genes sharing the same function or phenotype (see text). Per personal genome, the top row columns 2–7 describe the obtained top prediction from personal genome data and its properties. The Fold enrichment and FDR *q-value* are both reported by GREAT’s binomial enrichment test, fraction of relevant genes is the number of genes annotated for the phenotype (those listed in affected target genes) divided by all genes annotated with the phenotype. Column 8 highlights the matching personal medical phenotype. The bottom row for each personal genome spanning columns 2–7 provides exact quotes from references that confirm the link between the predicted and observed phenotypes (columns 2 and 8 for each personal genome).

	Personal genome based prediction						
Person	Affected phenotype	# of CoBEL loci	Fold	False Discovery Rate (Q-value)	Affected target genes	Fraction of relevant genes	Personal medical phenotype
**Stephen Quake**	abnormal cardiac output	57	2.00	1.69 x 10^−4^	ADRA1A, ADRA1B, ARSB, CACNB2, CDC42, CDH2, DDAH1, ELN, FXN, MLYCD, NPPA, NRG1, PDLIM3, PLN, PPARGC1A, PPARGC1B, RAF1, RXRA, TMOD1	58%	family history of ARVD/C and heart disease and presumed sudden cardiac death
	“Arrhythmogenic right ventricular dysplasia/cardiomyopathy is an inherited cardiomyopathy estimated to affect approximately 1 in 5,000 individuals. [. . .] The disease is frequently familial and typically involves autosomal dominant transmission with low penetrance and variable expressivity.” [21]						
**George Church**	preganglionic parasympathetic nervous system development	23	3.26	1.18 x 10^−4^	EGR2, HES1, HES3, HOXA1, HOXB1, HOXB2, PLXNA4, TFAP2A	80%	narcolepsy
	“… a non-secondary involvement of the autonomic nervous system in narcolepsy is strongly suggested” [22]						
**Misha Angrist**	epithelial cell morphogenesis	60	2.11	1.38 x 10^−5^	BASP1, BCL11B, BMP4, CTNNB1, EPB41L5, FZD7, GATA3, GDNF, GREM1, HEG1, IHH, PAX2, PAX8, SALL1, SIX2, WT1	59%	possible keratosis pilaris
	“The epidermis [in keratosis pilaris] demonstrates mild hyperkeratosis, hypogranulosis, and follicular plugging.” [23]						
**Rosalynn Gill**	decreased circulating sodium level (hyponatremia)	32	3.23	4.94 x 10^−6^	EDN1, NR3C2, SCNN1B, SCNN1G, SLC26A3, SLC4A4, TXNIP, WWOX	89%	hypertension
	“A sodium-conserving genome in the context of the contemporary high-sodium and low-potassium diet is maladaptive, with documented pathological and epidemiological consequences (ie, epidemic hypertension).” [24]						
**James Lupski**	regulation of oligodendrocyte differentiation	59	2.11	2.93 x 10^−5^	ASPA, BMP4, CTNNB1, CXCR4, DLX1, DLX2, HDAC2, HES1, HES5, ID2, ID4, LINGO1, OLIG2, PPARG, SHH, TCF7L2	73%	family history of patchy axonal polyneuropathy
	“Oligodendrocytes, the myelin-forming glial cells of the central nervous system, maintain long-term axonal integrity.” [25]						

The top enrichment for George Church, who suffers from narcolepsy, is “preganglionic parasympathetic nervous system development” (23 CoBELs, Q = 1.18 x 10^−4^). The autonomic nervous system is strongly suspected to be involved in narcolepsy [22]. Misha Angrist, whose personal reporting indicates possible keratosis pilaris, a follicular condition manifested by the appearance of rough, slightly red, bumps on the skin, has “epithelial cell morphogenesis” as his top biological process enrichment [23] (60 CoBELs, Q = 1.38 x 10^−5^). For Rosalynn Gill, who suffers from hypertension, the top enriched phenotype is “decreased circulating sodium level” (32 CoBELs, Q = 4.94 x 10^−6^). Sodium intake is strongly associated with hypertension [24]. Intriguingly, the top biological process enrichment we obtain for James Lupski, whose family has a history of axonal neuropathies in the peripheral nervous system (PNS) [20], is “regulation of oligodendrocyte differentiation” (59 CoBELs, Q = 2.93 x 10^−5^). Oligodendrocytes are the neuroglia that create the myelin sheath around axons in the central nervous system (CNS) and maintain long-term axonal integrity [25,26].

While a statistically significant functional enrichment from GREAT rejects the null hypothesis of uniformly random distribution of the CoBELs in the regulatory domains of the function-associated genes, it does not check whether there is an inherit bias in the distribution of conserved binding sites (eroded or not) in the regulatory domains of genes involved in the enriched functions. Thus to further assess the significance of our results we replaced every CoBEL with a random binding site prediction for the same transcription factor of same affinity and similar cross-species conservation. Using 10,000 random control sets, the likelihood of obtaining the functions reported in Table 1 as top prediction due to bias in the distribution of binding sites in the genome is low (Quake *P* = 3 x 10^−4^, Church *P* = 5.7 x 10^−3^, Angrist *P* = 4.8 x 10^−3^, Gill *P* = 1 x 10^−4^, Lupski *P* = 1.9 x 10^−3^, and combined P = 1.6 x 10^−15^). Significance remains high when we relax the requirement to recover each exact same term with matching any one of a broader group of 12–60 related functions as a top prediction (Quake *P* = 1.1 x 10^−3^, Church *P* = 1.3 x 10^−2^, Angrist *P* = 7.7 x 10^−3^, Gill *P* = 7.4 x 10^−3^, Lupski *P* = 6.5 x 10^−3^, and combined P = 5.2 x 10^−12^; see Materials and Methods).

While phenotypic data is not available for the 1,000 genomes project subjects [27], the availability of whole genome sequences for 1,094 individuals allows us to ask how unique are our top predictions for the five phenotyped individuals against a large background of controls. We asked whether the phenotype predictions were unique to a given personal genome by testing whether they rarely appeared in control individuals from the 1,000 genomes project, thereby testing the specificity of our screen. This control analysis was performed due to the inclusion of both common and rare variants in our analysis. We wanted to verify that enrichments observed in our five genomes were not dominated by common CoBELs shared with many other individuals.

**Fig 2:**
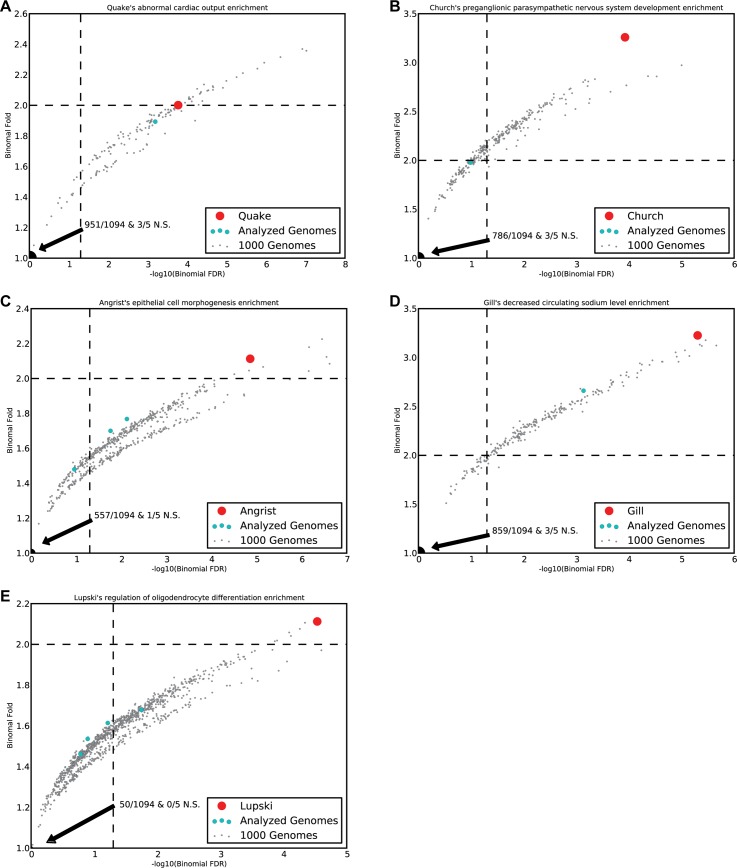
Enrichment distribution of hypothesized phenotypes in ‘control’ genomes. (A-E) Comparison of personal genome enrichments of 1,094 genomes from the 1,000 genomes project and the five genomes analyzed in this report. Dashed lines indicate GREAT’s default binomial fold (greater than or equal to two) and FDR (less than or equal to 0.05) significance thresholds. Lower left corner has the mass of genomes that were not significant by GREAT’s default hypergeometric FDR (less than or equal to 0.05). The red markers indicate an analyzed personal genome’s prediction is significant and distinguishes it from the 1,000 genomes cohort, indicating such associations do not spuriously appear at a high frequency in control individuals. Panel A indicates the enrichment of “abnormal cardiac output” is fairly common in the background 1,000 genomes cohort which is not unexpected since predisposition to mild forms of heart disease are common in otherwise normal populations.

**Fig 3:**
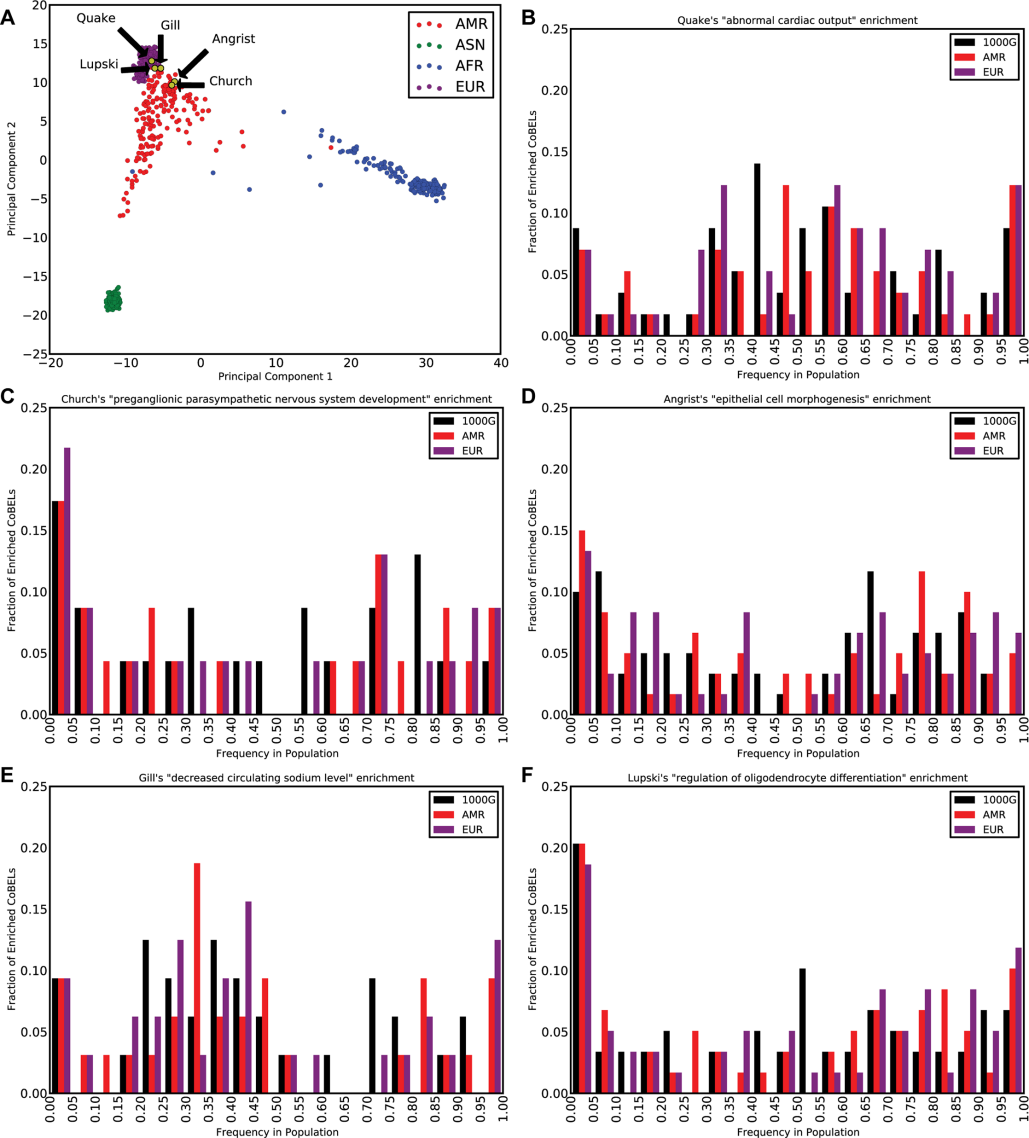
Frequency distribution of CoBELs in relation to population structure. (A) Principal component analysis (PCA) of the five genomes with respect to the genomes in the 1,000 genomes project, revealed clustering with the European population as expected. (B-F) Comparison of the five individuals's enrichment specific CoBEL frequencies in all 1,000 genomes data and in the two populations with which the five genomes cluster by PCA. Both this and additional frequency distribution analysis (see text) reveal that top CoBELs enrichment are composed of both common and rare variants as expected of low pathogenicity mutations that exert a noticeable effect only in aggregate. The similarity of the frequency distributions for the full 1,000 genomes and two sub-populations further suggests the lack of any population specific bias in our enrichments.

Thus, we computed the frequency of the observed enrichments in all control, un-phenotyped 1,094 genomes sequenced by the 1,000 genomes project [27]. We verified the CoBEL set size of the 1,094 genomes were comparable to those of the five analyzed genomes (min 6,121; European median 6,385), submitted the CoBELs to GREAT and noted top enrichments. Each one of our observed top enrichments for the five individuals had an occurrence rate less than 0.05 (S2A Table) and the enrichment’s p-value and fold statistics placed them as significantly removed from the 1,000 genomes cohort (Fig 2). Next, we performed PCA to verify that the five genomes analyzed in our study are both predominantly of the expected (European) ancestry, and not an outlier compared to the 1,000 genomes project data (Fig 3A). We then recomputed the occurrence rate for the enrichments using only the 381 European genomes and only the 181 admixed genomes to correct for any population specific enrichments. Again, all the enriched terms had an occurrence rate less than 0.05 (S2A Table). Since ontology terms in GREAT are related in a directed acyclic graph (DAG) structure, terms such as “abnormal cardiac output” (the Quake genome prediction) share similar gene sets to their umbrella term “abnormal cardiovascular output”, which a control patient from the 1,000 Genomes project may exhibit. To account for the case when two such related terms are predicted, we calculated the false discovery rate for a term by counting its broader group of related functions as well. Still, the occurrence rate for the findings remained less than 0.05 (S2B Table) when we repeated both the full 1,094 genomes, 381 European genomes and 181 admixed genomes calculations for the broader group of related functions, except for slightly higher p-values (up to 0.088) for the more common heart and hypertension disorders. Indeed, 8% of the un-phenotyped 1,000 genome subjects (who may themselves suffer or be predisposed to various complex diseases, especially the more common ones) had a top enrichment in the broader set of terms associated with hypertension, and 5% were similarly most enriched for a heart term.

Finally, we assessed the specificity of associating the CoBEL enrichments of five individuals with their medical histories (Fig 1C and S14 Table). This test was performed to verify that the predicted top enrichments were not so broad that they would match different medical histories and likewise that the individuals selected did not have such a broad range of disease phenotypes as to match different possible top enrichments. We defined an association matrix linking enrichment and medical history, with the phenotypes observed in the five individuals as rows, and top enriched terms in all as columns. A cell in the matrix would be marked “true” only where the enriched term (of any individual) is thought to be related to the etiology of the phenotype (of any individual; see Materials and Methods). One instance of this matrix was filled by a medical doctor based on their medical knowledge and training (S15 Table) and another instance was independently filled using a literature survey (S16 Table). The objective was to compute the chance of associating a set of five individuals with random medical histories with the observed enrichments using one of the two association matrices as the “gold” association. We generated 1,000 sets of five individuals with random medical histories composed of similar disease profiles and assessed the likelihood of being able to associate them with enrichments (see Materials and Methods). Successfully linking five random individuals with enrichments was highly significant using the association matrix generated by the medical doctor (*P* = 3.0 x 10^−3^) and by the matrix generated by literature survey (*P* = 3.0 x 10^−2^) suggesting our links between enrichment and medical histories are not just a function of the listed histories. The literature survey derived association matrix potentially offers a stricter null model since it includes associations that are currently research topics hinting at associations that may or may not become clinically relevant in the future.

Our CoBEL predictions are distinct from known GWAS associations. The 238 variant alleles that underlie all Table 1 predictions overlap a single, phenotype irrelevant, GWAS SNP, suggesting our method as a complementary method to discovering disease loci. While GWAS aims to find loci most likely to individually distinguish disease cohorts from matched controls, our method tries to identify the sum of both common and rare loci that can contribute to disease. GWAS is underpowered to find such combinatorial interactions. Similarly, none of the CoBELs responsible for the 238 variants intersect with a HGMD [28] disease variant (a large set of very rare, highly penetrant variants thought to individually trigger the underlying disease). When the overlap analysis is extend to include GWAS SNPs in possible linkage disequilibrium (LD), only two possible phenotype matches arise: “cardiac hypertrophy” associated [29] SNP rs3729931 for Quake, and “multiple sclerosis” (another demyelination disease [26]) associated [30] SNP rs882300 for Lupski. Indeed, nearly half the total number of CoBEL variant alleles we predict (7,115, 49%) are unique to only one of our five individuals. Similarly, for each of the five top function predictions in Table 1, of sixteen possible subsets (CoBELs shared or not with each of the other four individuals), the biggest contribution (17–34%) always comes from private sites (S1 Fig).

When the CoBEL frequencies are examined at the population level, Quake and Gill’s enriched CoBELs show higher population frequencies (Fig 3B and 3E) for their presumably more common enriched phenotypes of heart disease and hypertension. Conversely, Church, Lupski and even Angrist to a lesser extent, show more enriched CoBEL with low population frequencies (Fig 3C,3D and 3F). To examine the population frequency dependence of the CoBEL analysis, we restricted ourselves to rare CoBELs, defined as those with frequency less than or equal to 0.01 in the 1,000 genomes. None of our functional enrichments are significant for the rare CoBELs. Even when we increase the 1,000 genome frequency 10-fold to 0.1, only Angrist’s “epithelial cell morphogenesis” enrichment is rescued, albeit with diminished enrichment statistics (16 CoBELs, Q = 1.85 x 10^−2^) compared to the full set (60 CoBELs, Q = 1.38 x 10^−5^). This further corroborates that our enrichments are a combination of both common and rare variants.

## Discussion

The screen we perform is underpowered: we do not have the binding affinities of all human transcription factors or all functional (ancestral or not) binding sites; variant mapping may miss more complex gene regulatory mutations; and in particular our knowledge of phenotype to gene associations is far from complete. Additionally, we focus only on the top enrichment obtained rather than all enrichments to maintain the ability to test for statistical rigor of the associations. All these limitations, however, only reduce our power to detect true associations, but do not elevate the likelihood of false predictions. In contrast, by focusing on deeply conserved binding sites, we greatly increase the likelihood that their disruption carries a fitness cost. Indeed, considering that GREAT tests over 16,000 different biological processes or phenotypes (from “abdominal aorta aneurysm” to “zymogen granule exocytosis”), the links we obtain between genomic prediction and medical phenotype seem highly significant.

Our CoBEL predictions compliment known disease alleles. For example, a particular human leukocyte antigen (HLA) allele is found in a vast majority of narcolepsy patients who suffer from cataplexy, and is also common in narcolepsy patients who do not [31]. The affected Church genome is homozygous for a different HLA allele (see Supplementary Methods). Four GWAS SNPs, all with modest effect size (OR = 1.29–1.79) are currently associated with narcolepsy. Church carries two of these, but the other four unaffected genomes we analyze each carry 2–3 narcolepsy risk alleles as well, due to their common prevalence (see S3 Table).

The Quake genome was previously analyzed for coding and GWAS variants [18]. While no single strong mutation emerged, the sum of collected mutations was enough to assess heart disease as a relatively large risk. The evaluation process of the many personal variants however was biased towards genic variants and previously determined risk loci with a focus on explaining the family history of heart disease. The enrichment we obtain for cardiac output not only comes from novel, non-genic loci, it is also obtained in a completely agnostic fashion.

Our analysis is complementary to state of the art analyses that focus on searching for the primary disease causing variant by intersecting with known (predominantly coding) variant databases, exploring rare or novel coding or splicing variants in known disease associated genes and prioritizing coding candidate variants using computational tools such as SIFT [32], PolyPhen2 [33] and VAAST [34]. Few such tools exist for the non-coding genome, none of which to the best of our knowledge focuses explicitly on binding site disruption. Methods such as CADD [35] score the pathogenicity of non-coding variants, but train their model on positive sets only weakly enriched for deleterious non-coding mutations. Because the non-coding portion of the genome is so large (97%), and because most such tools do not aggregate mutations on functional or any other categories, most usage is restricted to splice variants or non-coding RNA. This is exemplified by the genome analyses performed by Lupski et al. [20] and Ashley et al. [18], for Lupski and Quake, respectively. Both works focused primarily on coding variants in known disease associated genes. They identified non-synonymous SNPs and searched for matches in known pathogenic variant databases such as HGMD [28] and OMIM [36]. When known disease variants were not identified, the search was expanded to include rare and novel variants in genes relevant to their patient (neuropathies in the case of Lupski et al. [20] and cardiovascular disease in the case of Ashley et al. [18]). Neither study pursued any potential gene regulatory mutations.

In addition to the enrichment obtained by our analysis, the accumulation of binding sites in our top enrichments is also revealing: First, each target gene in Table 1 is affected, on average, by more than three CoBELs, chipping away at the gene’s presumed regulatory robustness [37]. Second, Table 1 also shows that in all five cases, CoBELs affect a majority (58–89%) of all human genes annotated for said function/phenotype.

Together, our observations suggest the gradual erosion of gene regulation over both (human generation) time and (gene regulation) space, ultimately manifesting as medical history. These observations corroborate a long held notion that lineage accumulation of small deleterious mutations, even when combined with different lifestyles and environments, ultimately increase the likelihood of familial disease phenotypes [38]. Depending on the selection coefficient of these deleterious mutations and their genetic background, these mutations may eventually be swept out of the population, but are currently visible due to nonrandom human mating patterns and the relatively short timescales since erosion.

Our screen provides an exciting glimpse of the latent genetic load of human gene regulation contribution to personal medical histories. As our ability to characterize individual genetic load improves, so will our understanding of genome–environment interactions, and the thresholds that are crossed to trigger onset of human disease.

## Materials and Methods

### Transcription factor binding motif library

Our transcription factor binding motif library, represented as a position weight matrix (PWM), contains 917 unique high quality monomer and dimer motifs for 657 transcription factors from the UniPROBE [39], JASPAR [40], and TransFac [41] databases, secondary UniPROBE motifs, motifs from published ChIP-seq datasets and from other primary literature [16]. We included both monomeric and dimeric (where the TF complexes either with itself or with another TF) motifs to improve our sensitivity since previous work has found that complexes tend to have modified binding affinities [16].

### Personal genomes and medical history summaries

We downloaded variant calls mapped to the human reference assembly hg19 (GRCh37) from the UCSC genome browser [42]. The tables were pgQuake for Stephen Quake, pgChurch for George Church, pgAngrist for Misha Angrist and pgGill for Rosalynn Gill. The variants for James Lupski were downloaded from dbSNP [43] and processed to remove non-single nucleotide polymorphism and those that had ambiguous mapping to the reference genome. The medical history summaries for Stephen Quake and James Lupski were obtained from Ashley et al. [18] and Lupski et al. [20], respectively. Medical history summaries for the remaining individuals were obtained from their public profiles on the Personal Genome Project [19] website.

### Identification of conserved binding site eroding loci (CoBELs)

We identified conserved binding sites using the UCSC human reference assembly hg19 (GRCh37) based multiple alignment of 33 primates and mammals [42]. Binding site prediction was done by identifying conserved binding site matches using PRISM [14]. We chose the default PRISM thresholds of a minimum of five species preserving each site prediction, with the total phylogenetic (neutral) branch length [44] of the preserving species amounting to two substitutions per site or more. Additionally, we kept only the top 0–5,000 binding site predictions that had a conservation *p-value* less than or equal to 0.05. The conservation *p-value* was computed by comparing the binding conservation for (CpG preserving) shuffled versions of the motif in similarly conserved regions of the genome. All parameter settings we used have been previously optimized in the PRISM paper for predictive power [14], including against multiple ENCODE [6] datasets.

Next, we identified all the heterozygous or homozygous variants in an individual genome where the human reference (hg19) base is identical to the orthologous chimp (panTro2) base, and thus most likely human ancestral. We then found all human reference genome conserved binding sites affected by our individual specific variants. Of these we kept only sites where replacing the reference human (ancestral) base(s) with the individual derived variant(s) lowers binding affinity by five per cent or more. Binding affinity was computed using the MATCH scoring scheme [45]. Overlapping binding sites were combined to obtain our final set of conserved binding site eroding loci (CoBELs).

### Algorithm for calling CoBELs per individual

Define set of human conserved binding sites, TFBS ← PRISM(motif library)

For each individual with genomic variants V_i_:

    Intersect TFBS with V_i_ (using overlapSelect from UCSC genome browser)

    For each TFBS tfbs in intersection:

        Compute tfbs MATCH score difference between ancestral and variant D

        If D decreases the MATCH score more than 5% add binding site to set of CoBELs

    Run GREAT(set of CoBELs) -> Output Top Enrichment

The majority of the motifs used in our screen are public, and can be obtained, along with their predictions, from the PRISM website at PRISM.stanford.edu. A small fraction of motifs comes from the proprietary Transfac database. A list of these will be provided upon request. We also include all five CoBEL sets in S4–S8 Tables, which can be processed using GREAT at GREAT.stanford.edu to reproduce the results of Table 1 and S9–S13 Tables.

### Inferring statistically significant accumulation of CoBELs next to genes that share a function or phenotype

Each set of CoBELs was submitted to GREAT (for Genomic Regions Enrichment of Annotations Tool) v2.0.2 [9]. As explained in the main text, GREAT searches for statistically significant genomic regions (in this case CoBELs) accumulation in the regulatory domains of genes that share the same annotation. For this study, we used GREAT’s default regulatory domain definition: a constitutive 5,000 bases upstream and 1,000 bases downstream of a gene’s canonical transcription start site (TSS), extended up to the constitutive regulatory domain of the adjacent genes on either side, or up to one million bases. Significance was also defined using the default GREAT thresholds: 0.05 FDR threshold for both binomial and hypergeometric test and binomial fold greater than 2. These parameter settings have all been optimized for inference power in the original GREAT paper [9]. We queried the GO Biological Processes [46] and MGI Phenotype [47] ontologies allowing GREAT to test for possible enrichment of any of 16,054 different functions, using 1,140,682 gene to function mappings.

### Estimating the significance of our Table 1 enrichments against shuffles

#### Generating 10,000 random control sets for each individual

Each CoBEL is a binding site overlapped by the individual’s variants file (Fig 1B). In cases of overlapping binding sites, the site that sustained the greatest decrease in binding affinity was chosen. With the binding site mapping, 10,000 random size matched sets were generated by sampling for each CoBEL a random binding site that has an identical binding affinity and a cross-species excess conservation *p-value* within the same order of magnitude as the actual CoBEL.

#### Defining the sets of related terms

The set of related terms for those reported in Table 1 was obtained by using the ontology structure defined by GO Biological Processes [46] and MGI Phenotype [47]. Using the ontology defined relations, we identified more general terms (ancestors) of those in Table 1 and defined each set of related terms as one containing the ancestor and all descendant terms (including the term for Table 1 and dozens more).

For Quake, a set of 60 related terms was defined as a (null set) match using the ancestor term “abnormal blood circulation”. For Church, a set of 12 related terms was defined using “autonomic nervous system development”, for Angrist, a set of 22 related terms was defined using “epithelial cell development”, for Gill, a set of 57 terms was defined using “abnormal mineral homeostasis” and for Lupski, a set of 21 terms was defined using “regulation of gliogenesis”.

#### Computing p-values for null hypothesis tests–linking CoBELs with enrichment

The p-value for both null hypothesis tests was computed empirically by counting the number of times the top GREAT enrichment obtained using the random control sets was the same term reported in Table 1 (null hypothesis 1) or was in the set of related terms to the term in Table 1 (null hypothesis 2).

### Computing the occurrence of enriched terms in the 1,000 genomes

The CoBEL methodology was applied to each of the 1,094 genomes and the top enrichment satisfying the default GREAT filters in the GO Biological Processes and MGI Phenotype ontologies was tracked. For each of the enrichments highlighted for the five genomes analyzed in this report, the frequency of the enrichment in the full 1,094 genomes was computed. Additionally, the frequency of the enrichments in the 381 European (EUR) subset and 181 admixed (AMR) subset was measured since principal component analysis revealed that the five genomes analyzed in this report are closest to these two population subgroups (Fig 3A).

### Estimating the significance of our Table 1 enrichment-medical history associations

#### Generating 1,000 sets of five individuals with random medical histories

The mapping between each individual and their medical histories (S14 Table) was shuffled 1,000 times to creating 1,000 sets of five individuals with random medical histories–to ask the question–if we had obtained five individuals with random medical histories, what is the chance of linking them to the observed CoBEL enrichments. We required that the random individuals to have similar number of medical history entries each and for each medical history entry’s occurrence frequency to match that observed in the true set. We also required that 80% (55/68) of the pairings between individuals and medical histories be different to avoid creating individuals with medical histories that were too similar to those of the observed individuals.

#### Defining the medical history–CoBEL enrichment association matrix

Two independent association matrices were defined to link all observed medical histories and CoBEL enrichments. The rows of the matrix were all the medical histories reported for each of the five individuals and the columns of the matrix were the single top GREAT GO Biological Process and MGI Phenotype enrichments observed in each of the five individuals. The rows and columns were linked (to create a ground truth) once using a medical doctor’s training and separately by a literature survey. The first matrix (S15 Table) was linked by a medical doctor based on their medical knowledge. Their objective was to assess the possibility of a "medical history" due to mis-regulation of genes involved in "CoBEL enrichment" and/or "CoBEL enrichment" leads to/causes/implicates the organ system of the "medical history". The second matrix (S16 Table) was assigned independently by a different author using an in-depth survey trying to link all genes (columns) to all phenotypes (rows) using the current published medical and research literature. Neither matrix was refined by the other technique and both were used independently to test the promiscuity of linking one of the observed enrichments with one of the observed medical histories.

#### Computing p-values for null hypothesis test–linking enrichment with medical history

The p-value was computed empirically by counting the number of times the 1,000 random sets of five individuals with random medical histories were by chance associated with an enrichment using a given association matrix (S15 and S16 Tables).

### Enriched CoBELs overlap or linkage with GWAS SNPs

All SNPs from the NHGRI GWAS catalog [48] were downloaded from a build containing 8,967 records in hg19 (GRCh37) co-ordinates, and intersected with the set of enriched CoBEL variant alleles from Table 1. Quake, Angrist, Gill and Lupski had no overlaps. Church had a single, phenotype irrelevant, overlap with rs10808265 which is GWAS associated with pulmonary function decline [49].

To assess linkage disequilibrium (LD) between the enriched CoBEL variants and GWAS SNPs we used HapMap [50] rel27 LD data for the CEU (Utah residents with Northern and Western European ancestry) population. CoBEL variant alleles from Table 1 were mapped to HapMap by taking the HapMap provided hg18 (NCBI Build 36.1) coordinates, lifting them to hg19 using the UCSC browser liftOver utility [45] and intersecting with the CoBEL variants. Nearly half (49%, 112/227) the enriched variants sites could be mapped to HapMap probes. NHGRI GWAS SNPs were mapped to HapMap SNPs using rsIDs. A GWAS SNP and a CoBEL variant were called in LD, using a maximalist approach, if either D’ > 0.99 or r^2^ ≥ 0.8 or LOD (log odds) ≥ 2 between their matching HapMap probes.

### Enriched CoBELs overlap with HGMD SNPs

All enriched CoBELs from the five individuals were overlapped with the HGMD PRO 2015.2 set containing 130,218 disease mutations using overlapSelect from UCSC genome browser.

### Church genome human leukocyte antigen type

Over 90% of narcolepsy patients with cataplexy, and around 40% of narcolepsy patients without cataplexy carry human leukocyte antigen (HLA) type DQB1*06:02 [31]. The crystal structure of HLA-DQB1*06:02 (PDB ID: 1UVQ) [51] identified the representative amino acid haplotype of DQB1*0602 as F_9_G_13_L_26_Y_30_Y_37_A_38_D_57_ (subscript represents amino acid number in exon 2 of HLA-DQB1). Based on the variant call file, the haplotype present is George Church is different: Y_9_G_13_L_26_H_30_Y_37_A_38_D_57_. When we used BLAST to search the Church version of exon 2 against the IMGT/HLA Database [52], the allele closest to the observed haplotype was DQB1*06:03, not found associated with narcolepsy patients [53].

## Supporting Information

S1 Table GREAT enrichments for GWAS SNPs are congruent with GWAS phenotype. For GWAS phenotypes ranging from metabolic traits to cancer and Crohn’s disease, the set of non-exonic GWAS SNPs, from the NHGRI catalog, associated with the trait is most highly enriched for a GREAT annotation closely associated to the trait, suggesting gene regulatory mutations often congregate near key genes associated with each assayed phenotype. Per GWAS phenotype (column 1), top row columns 3–8 describe the obtained top GREAT prediction from the non-exonic loci associated with the GWAS phenotype and its properties. The Fold enrichment and FDR q-value are both from GREAT’s binomial enrichment test. Fraction of relevant genes is the number of genes annotated for the phenotype (those listed in affected target genes) divided by all genes annotated with the phenotype. Column 3 highlights the predicted GREAT term. The bottom row for each GWAS phenotype provides exact quotes from references that confirm the link between the observed and predicted phenotypes (columns 1 and 3, respectively).
(PDF)

S2 Table False Discovery Rate (FDR) of Enrichments using 1,000 Genomes Data. (A) CoBELs from control individuals from the 1,000 Genomes project were submitted to GREAT and the fraction of individuals with the same or (B) a related top enrichment to the top enrichment of the five analyzed genomes was computed. In all cases, less than 10% of control people, regardless of race, had the same or similar top enrichments as the five analyzed genomes.
(PDF)

S3 Table Narcolepsy associated SNPs. Narcolepsy associated GWAS SNPs are tallied for the five analyzed genomes, indicating Church, who has narcolepsy and two GWAS variants, is not unlike the other genomes in having 2–3 common narcolepsy variants. As such, our CoBEL based narcolepsy-associated prediction for Church comes from orthogonal means–namely ensemble effects of multiple CoBELs.
(PDF)

S4 Table The full set of CoBELs for the Quake genome. (XLSX)

S5 Table The full set of CoBELs for the Church genome. (XLSX)

S6 Table The full set of CoBELs for the Angrist genome. (XLSX)

S7 Table The full set of CoBELs for the Gill genome. (XLSX)

S8 Table The full set of CoBELs for the Lupski genome. (XLSX)

S9 Table The set of binding loci and predicted upstream factors for the Quake “abnormal cardiac output” prediction in Table 1. (XLSX)

S10 Table The set of binding loci and predicted upstream factors for the Church “preganglionic parasympathetic nervous system development” prediction in Table 1. (XLSX)

S11 Table The set of binding loci and predicted upstream factors for the Angrist “epithelial cell morphogenesis” prediction in Table 1. (XLSX)

S12 Table The set of binding loci and predicted upstream factors for the Gill “decreased circulating sodium level” prediction in Table 1. (XLSX)

S13 Table The set of binding loci and predicted upstream factors for the Lupski “regulation of oligodendrocyte differentiation” prediction in Table 1. (XLSX)

S14 Table The medical histories of each individual and the top enrichments for their CoBELs. These histories represent the most relevant disease phenotypes for each of individuals analyzed. The histories were obtained either from the original publications of their genomes and/or from the public database from which their genomes were downloaded.
(XLSX)

S15 Table The association matrix between all medical histories and enrichments as defined by a medical doctor. Each column represents an enriched biological process or phenotype. Each row represents a disease phenotype. An ‘X’ is placed only where the physician considers that biological process or phenotype can be linked with that disease phenotype.
(XLSX)

S16 Table The association matrix between all medical histories and enrichments as defined by a literature survey. Each column represents an enriched biological process or phenotype. Each row represents a disease phenotype. A value is placed only where the primary literature offers potential support for a causal connection between the two entities. Where the link is non-obvious, a Pubmed PMID offers a supporting reference.
(XLSX)

S1 Fig CoBELs shared across the five analyzed individuals. The number and distribution of CoBEL (conserved binding site eroding loci) SNPs for the enrichments listed in Table 1 for (A) Quake, (B) Church, (C) Angrist, (D) Gill, and (E) Lupski across the five personal genomes. Individual variants, colored red, make the largest contribution (17%-34%) across all five enrichments.
(TIF)
