# Supplementary material for: Erosion of Conserved Binding Sites in Personal Genomes Points to Medical Histories
Source: PLoS Comput Biol. 2016 Feb 4;12(2):e1004711. doi: 10.1371/journal.pcbi.1004711 (PMC4742230; doi:10.1371/journal.pcbi.1004711)
Supplement: S1 Table — For GWAS phenotypes ranging from metabolic traits to cancer and Crohn’s disease, the set of non-exonic GWAS SNPs, from the NHGRI catalog, associated with the trait is most highly enriched for a GREAT annotation closely associated to the trait, suggesting gene regulatory mutations often congregate near key genes associated with each assayed phenotype. Per GWAS phenotype (column 1), top row columns 3–8 describe the obtained top GREAT prediction from the non-exonic loci associated with the GWAS phenotype and its properties. The Fold enrichment and FDR q-value are both from GREAT’s binomial enrichment test. Fraction of relevant genes is the number of genes annotated for the phenotype (those listed in affected target genes) divided by all genes annotated with the phenotype. Column 3 highlights the predicted GREAT term. The bottom row for each GWAS phenotype provides exact quotes from references that confirm the link between the observed and predicted phenotypes (columns 1 and 3, respectively). (PDF) [file pcbi.1004711.s001.pdf]

S1 Table. GREAT enrichments for GWAS SNPs are congruent with GWAS phenotype.

GWAS Result		Genome based prediction					
GWAS Phenotype	# GWAS Associated Non-exonic SNPs	GREAT Enrichment	# Enriched SNPs	Fold	False Discovery Rate (Q-value)	Affected target genes	Fraction of relevant Genes
**Cholesterol, total**	40	abnormal circulating cholesterol level	11	10.55	3.16×10^{-5}	ABCA1, ABCG8, ANGPTL3, APOC1, ATXN2, GPAM, LDLR, LDLRAP1, LIPC, LIPG, PCSK9	4%
		"Our results identify several novel loci associated with serum lipids …" [1]					
**Fasting glucose-related traits (interaction with BMI)**	28	abnormal glucose homeostasis	12	6.26	8.32×10^{-4}	ABCB11, ADRA2A, CRY2, FOXA2, G6PC2, GCKR, GLIS3, GPAM, GRB10, MAPK8IP1, PCSK1, PDX1, PROX1, SLC2A2, TCF7L2	3%
		"We applied a joint meta-analysis approach to test associations with fasting insulin and glucose on a genome-wide scale. " [2]					
**Prostate cancer**	19	abnormal gland morphology	13	4.80	8.51×10^{-4}	ASCL2, BHLHA15, BIK, BMPR1B, CCND1, HNF1B, ITGA6, LMTK2, MSMB, MYC, NKX3-1, OTX1, SP4	1%
		"We identified seven new prostate cancer susceptibility loci on chromosomes 2, 4, 8, 11 and 22…"[3]					
**Crohn's disease**	62	immune system process	28	3.77	7.43×10^{-7}	CCL7, CD244, CEBPA, CYLD, ERAP2, FASLG, GPR65, ICOSLG, IKZF1, IL10, IL12B, IL18RAP, IL2RA, IRF1, IRGM, JAK2, LIF, LIME1, LNPEP, LY9, MAPKAPK2, NKX2-3, NOD2, PDGFB, PTGER4, PTPN2, SATB1, SBNO2, SLC7A10, SMAD3, SOX4, TNF, TNFSF11, TNFSF18	3%
		"Crohn's disease results from the interaction of environmental factors, including the intestinal microbiota, with host immune mechanisms in genetically susceptible individuals." [4]					
**Metabolic traits**	30	organic acid metabolic process	16	9.06	1.20×10^{-8}	AKR1C3, AKR1C4, CPS1, CYP4A11, ELOVL2, ELOVL6, FADS1, GCKR, HMGCS2, IVD, PHGDH, PRODH, SLC16A9, SLC22A4, SLC2A9, SLC7A6, SLCO1A2, SLCO1B1, UGT1A1	3%
		"We identified 37 genetic loci associated with blood metabolite concentrations…" [5]					

References

1. Teslovich TM, Musunuru K, Smith AV, Edmondson AC, Stylianou IM, Koseki M, et al. Biological, clinical and population relevance of 95 loci for blood lipids. Nature. 2010;466: 707–713. doi:10.1038/nature09270

2. Manning AK, Hivert M-F, Scott RA, Grimsby JL, Bouatia-Naji N, Chen H, et al. A genome-wide approach accounting for body mass index identifies genetic variants influencing fasting glycemic traits and insulin resistance. Nat Genet. 2012;44: 659–669. doi:10.1038/ng.2274

3. Eeles RA, Kote-Jarai Z, Al Olama AA, Giles GG, Guy M, Severi G, et al. Identification of seven new prostate cancer susceptibility loci through a genome-wide association study. Nat Genet. 2009;41: 1116–1121. doi:10.1038/ng.450

4. Franke A, McGovern DPB, Barrett JC, Wang K, Radford-Smith GL, Ahmad T, et al. Genome-wide meta-analysis increases to 71 the number of confirmed Crohn's disease susceptibility loci. Nat Genet. 2010;42: 1118–1125. doi:10.1038/ng.717

5. Suhre K, Shin S-Y, Petersen A-K, Mohney RP, Meredith D, Wägele B, et al. Human metabolic individuality in biomedical and pharmaceutical research. Nature. 2011;477: 54–60. doi:10.1038/nature10354
