# Supplementary material for: Erosion of Conserved Binding Sites in Personal Genomes Points to Medical Histories
Source: PLoS Comput Biol. 2016 Feb 4;12(2):e1004711. doi: 10.1371/journal.pcbi.1004711 (PMC4742230; doi:10.1371/journal.pcbi.1004711)
Supplement: S2 Table — (A) CoBELs from control individuals from the 1,000 Genomes project were submitted to GREAT and the fraction of individuals with the same or (B) a related top enrichment to the top enrichment of the five analyzed genomes was computed. In all cases, less than 10% of control people, regardless of race, had the same or similar top enrichments as the five analyzed genomes. (PDF) [file pcbi.1004711.s002.pdf]

S2 Table. False Discovery Rate (FDR) of Personal Genome Enrichments in 1,000 Genomes Data

A) Occurrence rate computed for each term

**Enriched Term**	**1,094 genomes**	**381 EUR genomes**	**181 AMR genomes**
abnormal cardiac output	17 (0.016)	11 (0.029)	4 (0.022)
preganglionic parasympathetic nervous system development	8 (0.007)	0 (0)	2 (0.011)
epithelial cell morphogenesis	3 (0.003)	1 (0.003)	1 (0.006)
decreased circulating sodium level (hyponatremia)	20 (0.018)	13 (0.034)	5 (0.028)
regulation of oligodendrocyte differentiation	1 (0.001)	1 (0.003)	0 (0)

B) Occurrence rate computed for each set of similar (ancestral + children) terms

**Enriched Terms (# terms with distinct gene sets) (# terms)**	**1,094 genomes**	**381 EUR genomes**	**181 AMR genomes**
abnormal blood circulation (60) (69)	58 (0.053)	27 (0.071)	16 (0.088)
autonomic nervous system development (12) (24)	16 (0.015)	4 (0.010)	5 (0.028)
epithelial cell development (22) 49)	6 (0.005)	2 (0.005)	3 (0.017)
abnormal mineral homeostasis (57) (69)	91 (0.083)	24 (0.063)	6 (0.034)
regulation of gliogenesis (21) (27)	28 (0.026)	15 (0.034)	5 (0.028)

* A distinct term is defined as one that is annotated with at least one unique gene compared to a set of terms.
