# Supplementary material for: Erosion of Conserved Binding Sites in Personal Genomes Points to Medical Histories
Source: PLoS Comput Biol. 2016 Feb 4;12(2):e1004711. doi: 10.1371/journal.pcbi.1004711 (PMC4742230; doi:10.1371/journal.pcbi.1004711)
Supplement: S3 Table — Narcolepsy associated GWAS SNPs are tallied for the five analyzed genomes, indicating Church, who has narcolepsy and two GWAS variants, is not unlike the other genomes in having 2–3 common narcolepsy variants. As such, our CoBEL based narcolepsy-associated prediction for Church comes from orthogonal means–namely ensemble effects of multiple CoBELs. (PDF) [file pcbi.1004711.s003.pdf]

S3 Table. Narcolepsy associated SNPs

Linkage study results			Allele Freq.	Ancestral (hg19+chimp)	Individuals				
Risk SNP	OR	Ref.			Church	Quake	Angrist	Gill	Lupski
**rs1154155-G**	1.69	[1]	0.23	T	T	T	**G**/T	**G**/T	T
**rs2305795-A**	1.29	[2]	0.63	G	**A**	**A**/G	G	G	**A** or **A**/G*
**rs5770917-C**	1.63	[3]	0.11	T	T	**C**/T	T	T	T
**rs2858884-A**	1.79	[4]	0.82	**A**	**A**/C	**A**/C	**A**	**A**	**A**

***dbSNP**[5] **record is ambiguous.**

References

1. Hallmayer J, Faraco J, Lin L, Hesselson S, Winkelmann J, Kawashima M, et al. Narcolepsy is strongly associated with the T-cell receptor alpha locus. Nat Genet. 2009;41: 708–711. doi:10.1038/ng.372

2. Han F, Lin L, Li J, Aran A, Dong SX, An P, et al. TCRA, P2RY11, and CPT1B/CHKB associations in Chinese narcolepsy. Sleep Medicine. 2012;13: 269–272. doi:10.1016/j.sleep.2011.06.020

3. Miyagawa T, Kawashima M, Nishida N, Ohashi J, Kimura R, Fujimoto A, et al. Variant between CPT1B and CHKB associated with susceptibility to narcolepsy. Nat Genet. 2008;40: 1324–1328. doi:10.1038/ng.231

4. Hor H, Kutalik Z, Dauvilliers Y, Valsesia A, Lammers GJ, Donjacour CEHM, et al. Genome-wide association study identifies new HLA class II haplotypes strongly protective against narcolepsy. Nat Genet. 2010;42: 786–789. doi:10.1038/ng.647

5. Sherry ST, Ward MH, Kholodov M, Baker J, Phan L, Smigielski EM, et al. dbSNP: the NCBI database of genetic variation. Nucleic Acids Res. 2001;29: 308–311.
