# Supplementary figures and images for: Erosion of Conserved Binding Sites in Personal Genomes Points to Medical Histories
Source: PLoS Comput Biol. 2016 Feb 4;12(2):e1004711. doi: 10.1371/journal.pcbi.1004711 (PMC4742230; doi:10.1371/journal.pcbi.1004711)

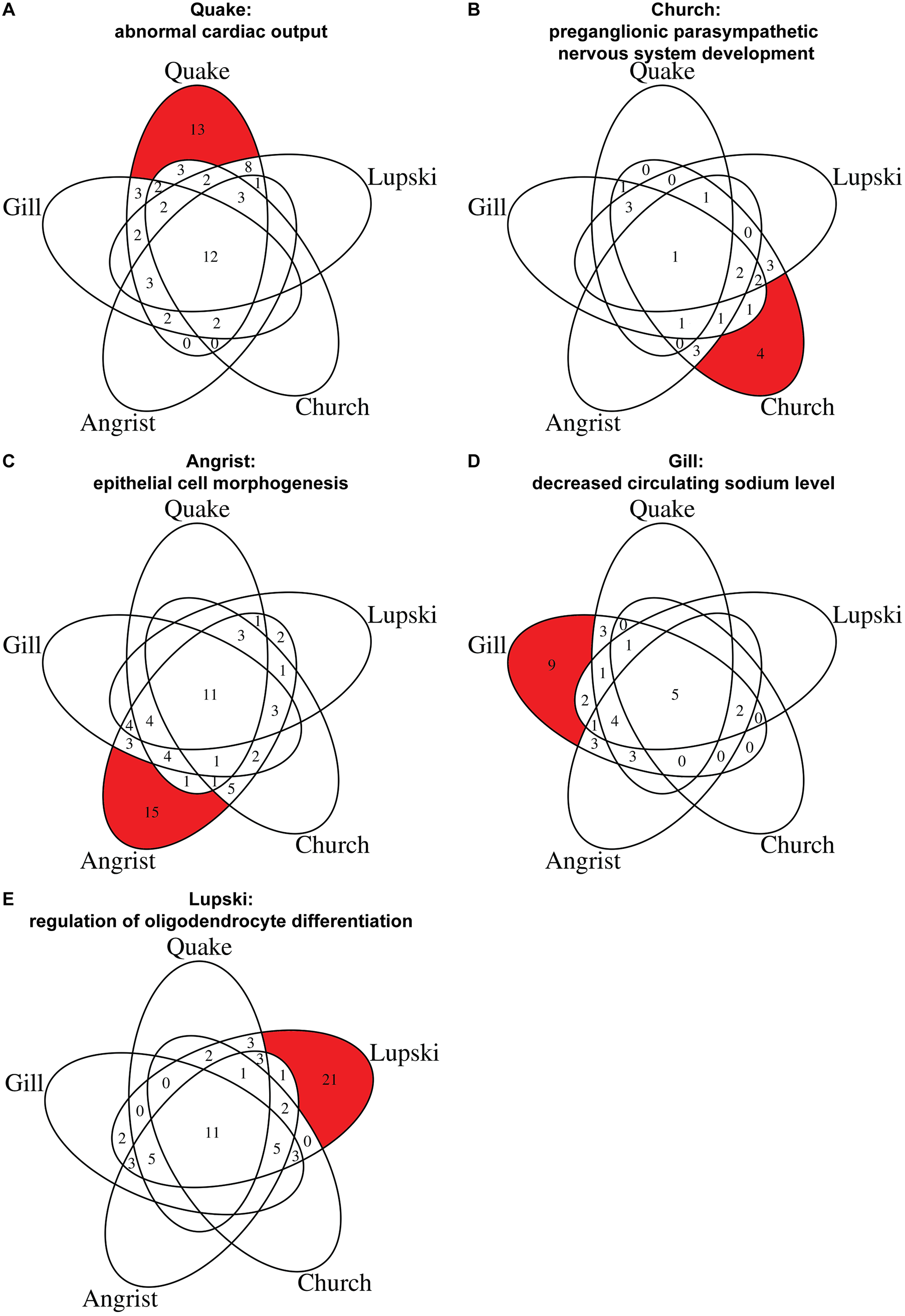

Supplement: S1 Fig — The number and distribution of CoBEL (conserved binding site eroding loci) SNPs for the enrichments listed in Table 1 for (A) Quake, (B) Church, (C) Angrist, (D) Gill, and (E) Lupski across the five personal genomes. Individual variants, colored red, make the largest contribution (17%-34%) across all five enrichments. (TIF) [file pcbi.1004711.s017.tif]
